# Foraging niche partitioning in sympatric seabird populations

**DOI:** 10.1038/s41598-021-81583-z

**Published:** 2021-01-28

**Authors:** Christina Petalas, Thomas Lazarus, Raphael A. Lavoie, Kyle H. Elliott, Mélanie F. Guigueno

**Affiliations:** 1 Department of Natural Resource Sciences, McGill University, Montreal, H3A 0G4 Canada; 2 Department of Biology, McGill University, Montreal, H3A 0G4 Canada; 3 Environment and Climate Change Canada, Québec, G1J 0C3 Canada

**Keywords:** Ecology, Behavioural ecology, Conservation biology, Ecology, Behavioural ecology, Conservation biology

## Abstract

Sympatric species must sufficiently differentiate aspects of their ecological niche to alleviate complete interspecific competition and stably coexist within the same area. Seabirds provide a unique opportunity to understand patterns of niche segregation among coexisting species because they form large multi-species colonies of breeding aggregations with seemingly overlapping diets and foraging areas. Recent biologging tools have revealed that colonial seabirds can differentiate components of their foraging strategies. Specifically, small, diving birds with high wing-loading may have small foraging radii compared with larger or non-diving birds. In the Gulf of St-Lawrence in Canada, we investigated whether and how niche differentiation occurs in four incubating seabird species breeding sympatrically using GPS-tracking and direct field observations of prey items carried by adults to chicks: the Atlantic puffin (*Fratercula arctica*), razorbill (*Alca torda*), common murre (*Uria aalge*), and black-legged kittiwake (*Rissa tridactyla*). Although there was overlap at foraging hotspots, all species differentiated in either diet (prey species, size and number) or foraging range. Whereas puffins and razorbills consumed multiple smaller prey items that were readily available closer to the colony, murres selected larger more diverse prey that were accessible due to their deeper diving capability. Kittiwakes compensated for their surface foraging by having a large foraging range, including foraging largely at a specific distant hotspot. These foraging habitat specialisations may alleviate high interspecific competition allowing for their coexistence, providing insight on multispecies colonial living.

## Introduction

The competitive displacement principle states that complete competitors, sharing identical overlapping ecological niches, cannot stably coexist in the same habitat at the same time^1–3^. Sympatric species must sufficiently partition their limiting resources, a phenomenon known as niche differentiation^4–7^. If we consider a species hypervolume (its ecological niche within an N-dimensional niche space), where each dimension corresponds to a state of the environment which allows a species to exist, niche differentiation implies that there is limited overlap within hypervolumes between coexisting species^5,8,9^. Niche differentiation permitting coexistence within the same habitat has been observed in a diverse range of taxa, from plankton to large carnivores^10–13^. Seabirds are intriguing in this regard because they often form large island colonies of breeding aggregations composed of several populations of species with seemingly overlapping diets (consisting largely of small fish) and foraging areas^14,15^.

While multi-species seabird colonies offer advantages such as defense against predators^16^, they also increase the chance for high interspecific competition due to similarities in many life-history traits^17–19^. This competition may peak during the breeding season when there are increased demands for food because individuals are restricted to forage within a limited range of the colony while needing to supplement themselves and their young^20,21^. In some multi-species seabird colonies, interspecific competition for nesting space may regulate the population size^18^, whereas for others that differentiate in nesting location may have competition occurring primarily at sea^22–24^.

Recent biologging tools have been used to show how colonial seabirds segregate components of their foraging strategies across space^17^ and prey choice^25^, leading to reduced interspecific competition^26–31^. Such segregation is associated with divergence in morphology potentially driven by ‘the ghost of competition past'^14,32,33^. That is, competition at the time of contact between two species in the past may have led to selection for morphologies (and, thus, foraging behaviour) that minimize competition. Current morphology (i.e. wingspan, mass, wing area) determines travelling distances and depths; flight costs increase with wing-loading, while dive depth increases with body mass due to higher oxygen stores^14,34,35^. Seabirds also vary morphologically in their bill depths which plays a role in determining the prey species, sizes, and numbers they can handle^36,37^. Single prey loaders should forage closer to the colony than multiple prey loaders due to the constraint of transit time^38^.

We examined whether and how niche partitioning occurs in four seabird species that breed sympatrically in the Gulf of St-Lawrence in Eastern Canada^39^ to understand the ecological processes that may partially alleviate the competition associated with colonial living. Specifically, we investigated diet using direct field observations of prey items carried by chick-rearing adults and spatial niches using GPS-tracking. At the Mingan Archipelago, three diving auk species, the Atlantic puffin (*Fratercula arctica*), razorbill (*Alca* torda), common murre (*Uria aalge*), as well as a non-diving pelagic larid species, the black-legged kittiwake (*Rissa tridactyla*), nest in close proximity with overlapping incubation and chick-rearing breeding stages while feeding mainly on forage fish^39,40^. We studied spatial foraging characteristics on incubating razorbills, murres and kittiwakes on Île à Calculot des Betchouanes (Betchouanes) using GPS-tracking and visual diet composition during chick-rearing on bill-loading species (puffins, razorbills and murres) on Betchouanes and Île de la Maison. These species have low competition for nest sites; puffins breed exclusively in burrows, razorbills and murres breed in rock crevices, and kittiwakes nest on cliff ledges. The species vary in terms of morphology determining their prey loading, flying, and diving capabilities which may constrain each species’ foraging niche. Bill depth morphology will determine the amount and size of prey a species can load in a foraging trip during the chick-rearing stage when they bring back prey within their bills to their chicks. Murres are single prey loaders while the other species are multiple prey loaders. We expect that single prey loading murres would forage the largest sized prey while few prey loading razorbills would load an intermediate size and multiple prey loading puffins would be foraging the smallest prey items. Murres also have the highest mass and wing-loading (mass per unit area of the wing), followed by razorbills, puffins, and kittiwakes, respectively^41^. Thaxter et al. showed that murres forage closer to the colony than razorbills due to their higher wing-loading, but because the study happened during the species’ chick-rearing stage, the effect may be partly due to murres being single prey loaders^33^. By studying seabird foraging movements during incubation, we eliminated the confounding effect of prey loading. That is, during incubation all birds can forage on all suitable fish, whereas during chick-rearing single prey loaders are constrained to bring a single prey back to their offspring, limiting foraging range. We expected that murres would forage closest to the colony, followed by razorbills and kittiwakes, respectively.

These trade-off differences in prey loading combined with birds’ flight and diving capabilities during foraging trips may have evolved to differentiate seabird niches to alleviate high interspecific competition and allow for multispecies colonial living^29,38,42,43^. Gaining insight on multispecies colonial living by understanding the link between foraging behaviour and population parameters could inform researchers on population sensitivity to environmental disasters. This could allow us to predict from an applied conservation perspective where seabirds are at sea and how they adequately respond to an environmental emergency^44^. Seabird communities have been tracked in most regions of Canada^19,28,45^, however, we are unaware of any published tracking studies in Quebec, and so we also provide the first fine-scale tracking information on seabird distributions along Canada’s longest provincial shoreline.

## Results

### GPS analysis

**Figure 1:**
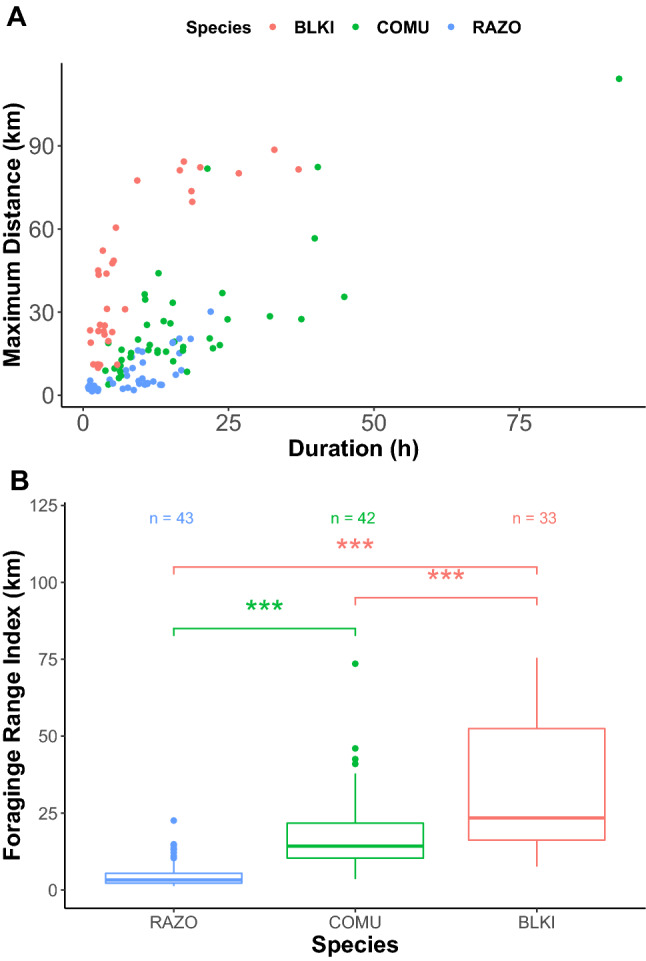
(**A**) Maximum distance travelled (km) during a trip for all foraging trips against the duration of the trip (h). (**B**) Foraging range indexes for foraging trips of razorbills (RAZO), murres (COMU), and kittiwakes (BLKI). n refers to total number of foraging trips for each respective species.

We analyzed the spatial dimension using GPS results from three incubating seabird species on Betchouanes island including razorbills, kittiwakes, and murres. We installed 11 GPS units on razorbills and recovered 4, 23 GPS units on kittiwakes and recovered 6, and 16 GPS units on murres and recovered data from 12. From this we obtained 122 complete foraging trips within the incubating breeding state (Supplementary Table S1). Kittiwakes travelled farthest, followed by murres and razorbills. The average duration of a foraging trip for each species were: 8.5 ± 0.9 h for razorbills, 17.7 ± 2.4 h for murres, and 8.6 ± 1.6 h for kittiwakes (Fig. 1). Further, the Foraging Range Index (FRI) of each species provides an estimate of the average distance (km) from the colony that the birds are foraging (see methods). FRIs differed among all three analyzed species tested (Kruskal–Wallis test: $\chi$^2^ = 65.6, df = 3, *p* < 0.001) (Fig. 1). FRIs differed between kittiwakes and both razorbills and murres (Wilcoxon test: razorbills: W = 1356, *p* < 0.001; murres: W = 1002, *p* < 0.001), and between murres and razorbills (Wilcoxon test: W = 1623, *p* < 0.001). These differences in FRI were found even after accounting for individual as a random effect (LME, F(2, 15) = 18.27, *p* < 0.001).

**Figure 2:**
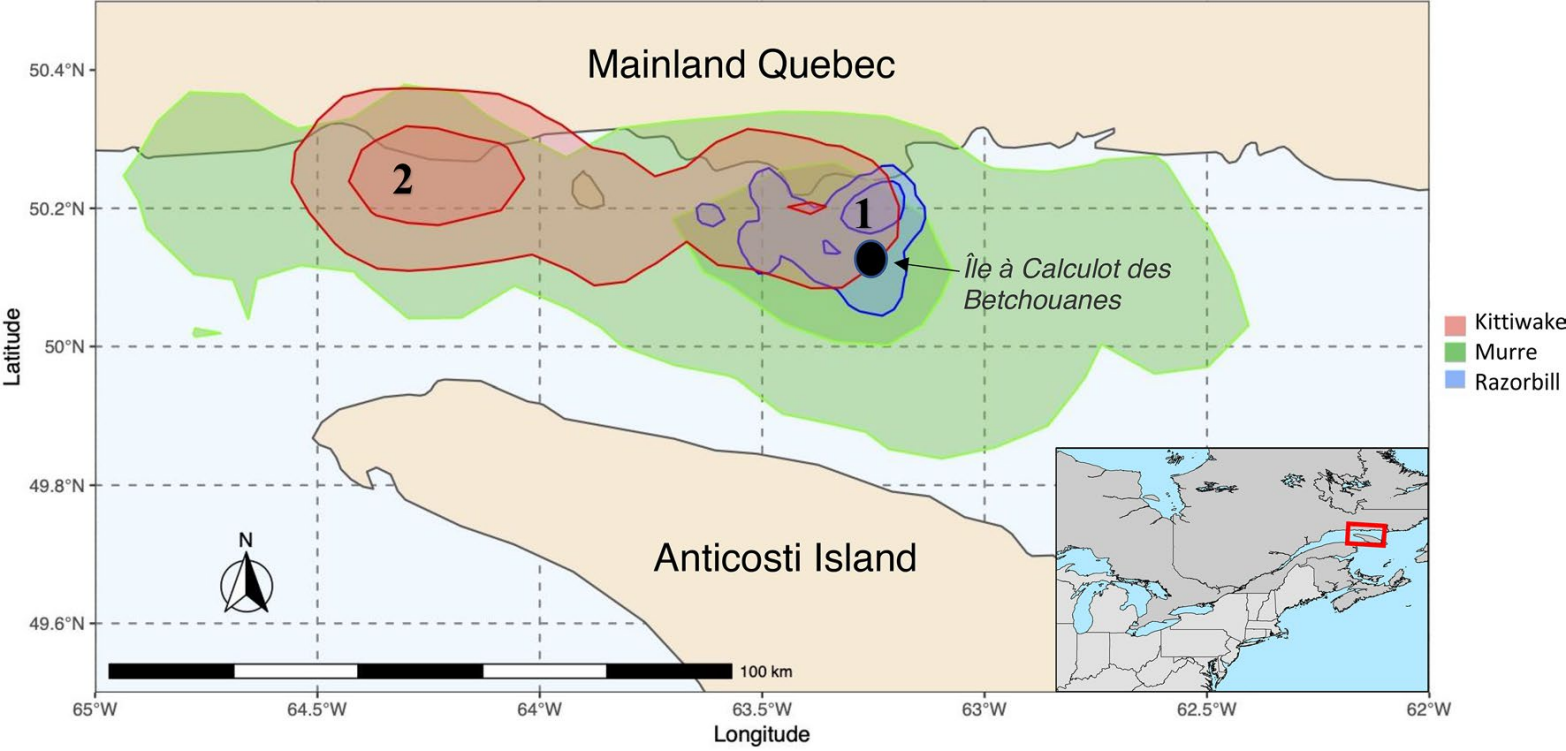
Utilization distribution contours (50, and 95% UD) of foraging locations of three species breeding on Île à Calculot des Betchouanes, in the Mingan Archipelago, Northern Quebec. Numbers indicate hotspot regions. Note that there are surrounding islands around Île à Calculot des Betchouanes that are too small to be depicted on map.

There was spatial segregation of all utilization distribution (UD) foraging ranges between all three species tested (Fig. 2). We calculated Bhattacharyya’s affinity (BA) indices for each species overlap comparison and obtained values ≤ 0.5, suggesting that the probability of foraging overlap was low between all species. Similarly, obtained UDOI values were ≤ 0.1 in all cases except between the home range (95% contours) between murres and kittiwakes (UDOI > 0.3), while their core foraging area (50% contours) overlapped considerably less (UDOI = 0.1, Table 1). Our randomization procedure (see methods) yielded all BA and UDOI indices to be significantly (*p* < 0.001) lower than the null expectation for 25%, 50%, 75% or 95% UDs (Supplementary Table S2).

**Table 1:** The estimated overlap (utilization distribution overlap index: UDOI and Bhattacharyya's affiniy: BA) in utilization distributions (UD %s) between three seabird species.

UD (%)	Black-legged Kittiwake/Razorbill		Black-legged Kittiwake/Common Murre		Razorbill/Common Murre	
	Observed BA	Observed UDOI	Observed BA	Observed UDOI	Observed BA	Observed UDOI
25	0	0	0	0	0	0
50	0	0	0.0545	0.003	0.1378	0.019
75	0.0859	0.008	0.3068	0.106	0.2479	0.062
95	0.2576	0.088	0.5291	0.386	0.4036	0.173

**Figure 3:**
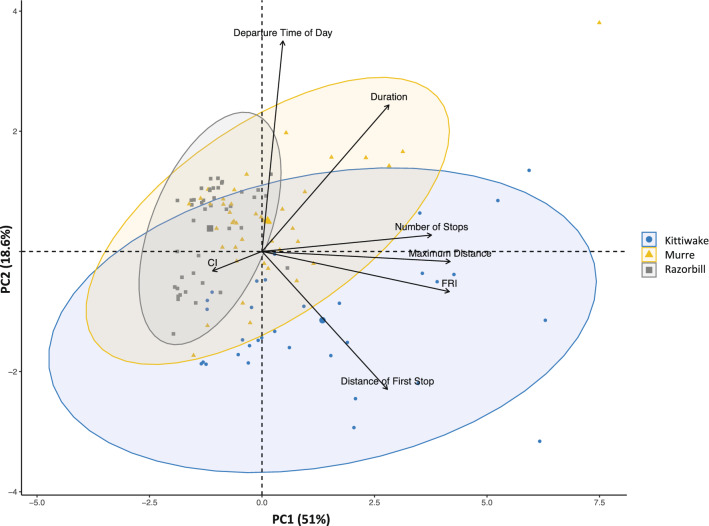
Principal Component Analysis (PCA) for seabirds in the Mingan Archipelago based on the following metrics: departure time from colony, maximum trip distance, duration of each trip, Foraging Range Index (FRI), distance at which bird first stops in foraging trip, number of patches visited, and commuting index (CI).

The foraging space differed between razorbills, murres, and kittiwakes (Fig. 3). There was a difference between the species tested along PC1 and PC2 (Kruskal–Wallis tests: $\chi$^2^ = 43.9, *df* = 2, *p* < 0.001; $\chi$^2^ = 42.9, *df* = 2, *p* < 0.001, respectively). A pairwise comparison of all three species showed, that they were significantly different from each other along the PC1 axis (Wilcoxon test: murre-kittiwake: W = 901, *p* = 0.02; razorbill-kittiwakes: W = 1247, *p* < 0.001; murre-razorbills: W = 1514, *p* < 0.001). The kittiwakes were significantly different than razorbills and murres along the PC2 axis (Wilcoxon test: W = 165, *p* < 0.001; W = 153, *p* < 0.001, respectively). Nonetheless, the foraging space of razorbills was almost entirely included within the foraging space of murres, which in turn was included within the foraging space of kittiwakes.

### Diet

We performed direct field observations on bill-loading auk species: puffins, murres, and razorbills during the chick-rearing breeding stage. All three seabird species were included at Betchouanes and two at Ile de la Maison (razorbills and puffins). Bird species foraged on significantly different prey species (Table 2) on both Betchouanes (puffins-razorbills: χ^2^ = 25. 8, *df* = 2, *p* < 0.001; puffins-murres: χ^2^ = 102.2, *df* = 9, *p* < 0.001; and murres-razorbills: χ^2^ = 117.3, *df* = 8, *p* < 0.001) and Île de la Maison (puffins-razorbills: χ^2^ = 22.6, *df* = 2, *p* < 0.001) (Supplementary Fig. S1). On Île de la Maison, we almost exclusively observed capelin (*Mallotus villosus)* and sandlance (*Ammodytidae* sp.). On Betchouanes, more varied diets were observed for all species, but this was mainly due to the presence of murres, which had a more diverse diet than other species. The murre diet was dominated by capelin, sandlance and gadids, while razorbills and puffins primarily foraged on sandlance and capelin. Murre diet also included a number of rarer species. Exclusively on Betchouanes, puffins diet included eelpout (*Gymnelus hemifasciatus*) and razorbill diet included Atlantic herring (*Clupea herengus*). The number of prey items loaded within a single foraging trip varied significantly between the two islands for both razorbills ($\chi$^2^ = 8.8, *df* = 1, *p* = 0.003) and puffins ($\chi$^2^ = 17.8, *df* = 2, *p* < 0.001) with more sandlance and fewer capelin on Île de la Maison. Four regurgitations were observed on Betchouanes from four kittiwakes, which exclusively consisted of capelin and sandlance.

**Table 2:** Frequency of occurrence, total numerical abundance and mass percent of diet for three seabird species breeding on islands in the Mingan Archipelago.

	Overall diet composition					
	Frequency of occurrence		Numerical Abundance		Mass	
	*n*	(%)	*n*	(%)	(g)	(%)
**Atlantic Puffin**						
Capelin	**60**	**41.09**	**193**	**40.46**	**687.0**	**58.18**
Sandlance	66	45.20	190	39.83	301.41	25.53
Larval Sp	19	13.01	92	19.29	184	15.58
Unknown	1	0.68	2	0.42	8.35	0.71
Total	146	100	477	100	1180.77	100
**Razorbill**						
Capelin	18	22.22	61	25.10	447.49	36.71
Sandlance	**58**	**71.60**	**165**	**67.90**	**628.76**	**51.57**
Atlantic Herring	2	2.47	9	3.70	76.03	6.24
Unknown	3	3.70	8	3.29	66.83	5.48
Total	81	100	243	100	1219.11	100
**Common Murre**						
Capelin	**165**	**50.92**	**165**	**50.92**	**2259.74**	**61.24**
Sandlance	42	12.96	42	12.96	247.71	6.71
Atlantic Cod	9	2.78	9	2.78	104.91	2.84
Gadidae Sp	36	11.11	36	11.11	428.14	11.60
Daubed Shanny	6	1.85	6	1.85	30.88	0.84
Stichidae Sp	11	3.39	11	3.39	175.59	4.76
Rock Gunnel	5	1.54	5	1.54	37.66	1.02
Atlantic Hagfish	1	0.31	1	0.31	0.05	0.001
American Shad	2	0.62	2	0.62	20.86	0.56
Atlantic Herring	14	4.32	14	4.32	127.99	3.47
Arctic Shanny	2	0.62	2	0.62	4.06	0.11
Pholidae Sp	1	0.31	1	0.31	1.65	0.04
Unknown	30	9.26	30	9.26	250.62	6.79
Total	324	100	324	100	3689.88	100

Given the large colony sizes (see “Materials and methods”), it is unlikely that the same individual was incorporated twice. However, because individuals were not marked, we cannot know for certain.

**Figure 4:**
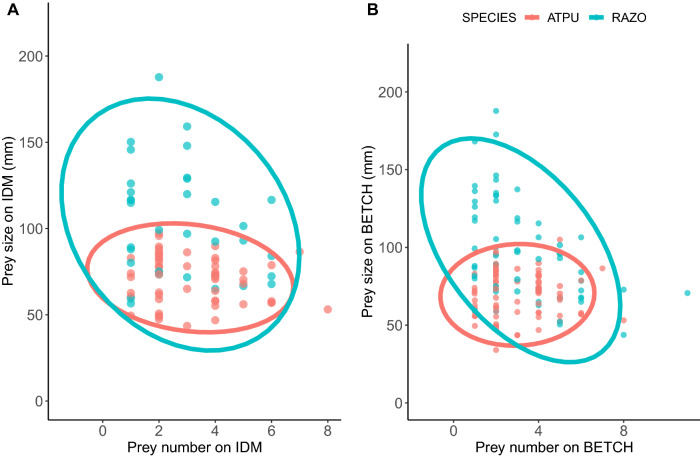
Prey size vs. prey number for both Atlantic Puffins (red) and Razorbills (blue) (**A**) on Île de la Maison (IDM), (**B**) on Betchouanes (BETCH). Each point represents one foraging observation. Figure includes feeding observations of capelin and sandlance.

The number of prey items loaded within the bills of species varied. Puffins and razorbills returned with 3.1 ± 0.1 SE and 3.0 ± 0.2 SE prey items, respectively, while murres strictly carried a single prey upon returning from a foraging trip. The numbers of prey loaded on Île de la Maison and Betchouanes varied for both puffins and razorbills. On Île de la Maison, prey loading was significantly different between puffins and razorbills (Quasi-Poisson test: t = − 3.2, *sd* = − 0.5, *p* = 0.002) but was not significant on Betchouanes (Quasi-Poisson test: t = 0.8, *sd* = 0.08, *p* = 0.4). Razorbills brought fewer prey on Île de la Maison than on Betchouanes, while puffins loaded fewer prey from a single trip on Betchouanes than on Île de la Maison (Wilcoxon tests: W = 1334.5, *p* = 0.02; W = 4592.5, *p* = 0.05, respectively) (Fig. 4).

The size of prey between all species varied significantly (Kruskal–Wallis test: $\chi$^2^ = 261.9, *df* = 2, *p* < 0.001; Wilcoxon test: puffins-murres: W = 1309, *p* < 0.001; puffins-razorbills: W = 1758, *p* < 0.001; murres-razorbills: W = 16,175, *p* < 0.001). Murres were foraging on average the largest fish sizes (116.4 ± 1.5 mm), the puffins, the smallest (64.1 ± 1.2 mm). The razorbills were foraging on average intermediate sizes of roughly 105.1 ± 4.02 mm.

We analyzed prey number in relation to prey size for both puffins and razorbills on both islands (Fig. 4). On Betchouanes, prey size decreased as number increased for razorbills (F-statistic test: F-statistic: *p* < 0.001, 5.192 on 1 and 140 *df*), and for every additional prey foraged within a single trip the size decreased by 7.372 mm.

## Discussion

Highly competitive environments, like large colonies of seabird species, should favor the evolution of adaptations to reduce niche overlap as a mechanism allowing for coexistence^4,46^. We found that the seabird species at the Mingan Archipelago differentiated in their spatial and dietary foraging characteristics, which may alleviate competition and permit multi-species colonial living (Table 3).

**Table 3:** Foraging and diet characteristics of the four studied species in the Mingan Archipelago.

	Average foraging distance (FRI, km)	Average flight duration (h)	Average number of prey items loaded	Average size of prey items (mm)	Total number of foraged prey species
Atlantic Puffin			3	64	5
Razorbill	5.3	8.5	3	105	4
Common Murre	18.4	17.7	1	116	13
Black-legged Kittiwake	35.3	8.6			

There was little overlap in utilization distributions, where UDOI and BA values were relatively low indicating segregation in foraging areas (Table 1). Razorbills forage relatively close to the colony compared with murres and kittiwakes (Figs. 1, 2). In Scotland at the Isle of May, razorbills tend to fly farther, and favour diving in relatively shallower water away from the colony, compared to murres^47^. Murres have 30% higher wing loading than razorbills leading to predictions that murres dive deeper and forage closer than razorbills who forage farther from the colony^43,48^. However, our data revealed that while both species had similar commuting behaviour, making many stops during a single foraging trip, incubating murres were typically foraging much farther than incubating razorbills and appeared to forage a wide range of habitats and prey species (Fig. 2, Table 2). One explanation for the difference is that murres are exclusively single prey loaders while razorbills can return with many prey items, and so we would expect that chick-rearing (but not incubating) murres would make many, short trips near the colony to feed their offspring because they cannot bring back many prey items all at once. Thus, murres are flying farther distances during the incubation because they are larger, and therefore fly faster and need to consume more energy per day but less often. There was more periodicity in foraging in auks than the kittiwakes, with auks leaving to forage later in the day than kittiwakes (Fig. 3). Also, spatial and dietary results further suggest that razorbills occupy a small niche space within the larger and more variable murre niche with respect to (1) their foraging areas (Fig. 2) and (2) their prey species preferences (Table 2). These razorbills seem to be more selective in the prey species foraged (primarily capelin and sandlance) while murres show greater flexibility in their prey choice (13 prey species foraged on). Hence, at the Mingan archipelago the studied razorbills may be partitioning aspects of their ecological niche by being more selective and less variable in their feeding habitats and prey choice than murres, which in turn are more flexible in their ecological niche.

Kittiwakes, like murres, were variable within their feeding habitats and foraged the farthest distances from the colony than other species. Kittiwakes tended to forage at particular locations, unlike murres (Fig. 2). Perhaps kittiwakes are travelling these longer distances to forage in specific patches that have predictable high densities of prey because they expend the least energy for flight (lowest wing loading) and can intake high energy loads (multi-prey loaders). Therefore, these kittiwakes, being surface feeders, may be compensating for relatively lower food availability near the colony by increasing their foraging range and distance from the colony and allocating more of their time to foraging in locations of higher prey densities^49^.

Murres were contained largely within the horizontal foraging space of the kittiwakes, where both species had relatively similar utilization distributions with two main hotspots (Fig. 2): 1: near the colony (a hotspot shared among all species) and, 2: ≈ 60 km west of Betchouanes. The second hotspot is a freshwater plume input to the marine water. Elsewhere, these convergent fronts aggregate prey^50^ and simultaneously attract marine predators, like seabirds^51^. This overlap in habitat distribution at this particular hotspot between the murres and kittiwakes may be overcome within the vertical dimension, by differing diving depths which has been shown to play an important role in niche segregation^43^. Indeed, murres have the highest diversity, including many benthic prey items, suggesting that they are, unlike all other species studied here, able to access deeper seafloor.

Auks dive to different depths within the water column^52^, exploiting different niches, and their activity underwater while chasing prey also differs^53^. Razorbills and puffins were observed to be multi-prey loaders, despite some studies finding razorbills to strictly load a few prey items at a time^40,48^. Future studies should gain insight on the foraging depths of each species while also conducting isotopic analysis on captured and recaptured individuals which could reveal whether there is niche differentiation in the vertical dimension and whether species like razorbills are able to saturate themselves while remaining close to the colony without having to travel farther distances, respectively.

Niche differentiation implies that there is limited overlap within ecological niches between coexisting species. Past competition could have played a role in producing the interspecific variation present in species’ ability to exploit habitats differently, permitting for their coexistence. The seabirds analyzed in the Mingan Archipelago partly may be overcoming such interspecific competition by segregating the dietary and spatial dimensions of their hypervolume (Table 3). Although there were some similarities in foraging hotspots, we observed clear species-specific foraging differences in diet, such as prey species, size and number. Whereas puffins and razorbills may forage multiple smaller prey items (Fig. 4) that were readily available to them closer to the colony, murres selected larger more diverse prey which were accessible due to their deeper diving capability and larger bills, while the kittiwakes compensated for their shallow surface dives by foraging largely at a specific distant hotspot. These foraging habitat specialisations may be a consequence of interspecific competition and ultimately providing further insight on multi-species colonial living.

## Materials and methods

### GPS tracking

Data was collected in 2019 at the Mingan Archipelago colony (Gulf of St-Lawrence in Northern Quebec, Canada) at two sub-colonies separated by ≈ 70 km; Betchouanes (50°11′ N, 63°13′ W) and Île de la Maison (50°13′ N, 64°12′ W). Betchouanes is a small (≈ 10.22 ha, maximum altitude ≈ 10 m) migratory bird sanctuary island situated ≈ 2.6 km east of the mainland covered by grasses and hosts various seabirds including three auk species colonies: Atlantic puffin (population size: 468 individuals), common murres (724 individuals), razorbills (1323 individuals) and a larid species; the black-legged kittiwake (252 individuals)^39^. Île de la Maison is a small privately-owned island (≈ 4.63 ha, maximum altitude ≈ 4 m) situated ≈ 4.8 km east from the mainland. The topography is characterized by a low central plateau with sparse vegetation surrounded by sand where puffins breed in burrows on the outskirts of the plateau and razorbills intermittently within abandoned puffin burrows and small boulder areas.

GPS loggers were attached to incubating breeding adults on Betchouanes and Île de la Maison between the 10th of June and 13th of July 2019 (Supplementary Table S1). All GPS loggers were attached onto the lower back feathers of the birds using Tesa tape and tie wraps except for the kittiwake, which were either attached onto the lower back feathers or the four central rectrices. These loggers corresponded to less than 3% of the body mass of all GPS-equipped species. The URIA-loggers allowed us to download the tracking data without having the necessity to recapture the bird by installing a base station fitted with a solar panel that would receive tracking data wirelessly whenever a GPS-equipped bird would return to their nest. The CatLog2-loggers were waterproofed in heat-shrink plastic tubing and were removed from the birds at recapture between 2 and 7 days after initial deployment. The CatLog2 and URIA -loggers were programmed to record latitude and longitude every 5 and 15 minutes, respectively.

Several techniques were used to capture and recapture the seabirds on both islands. For razorbills, we installed wooden nest boxes on both islands within areas where they would naturally breed which were equipped with an automated closing door that would shut by a remote trigger. Puffins were mostly captured by hand within their breeding burrows. Murres were captured within their boulder breeding sites using either our hands or a leg hook. The kittiwakes were captured using mist nets, noose matts (recapture only), a noose pole, and hand nets. For all captured and recaptured GPS-equipped birds we measured bill size using calipers and weight using a gravity scale. All work was carried out in compliance with the ARRIVE guidelines, specifically as approved by the Eastern Wildlife Animal Care Committee, Environment and Climate Change Canada (#19RL01) and followed all relevant guidelines and regulations for working with live vertebrates.

### Diet sampling of auk species

We conducted feeding watches on all three bill-loading auk species during the chick-rearing phase in order to investigate differences in diet^40,54^ (Supplementary Table S3). No feeding observations were made for the kittiwake because they regurgitate their prey instead of carrying it within their bills. On both islands, a blind was installed near the vicinity of the major breeding sites of GPS-equipped birds. Eight total feeding watches were conducted (4 on each island) where 1–3 observers recorded prey-carrying seabirds during 4–6-h shifts from the 1st to 12th of July 2019. For each bird observed returning to its nest carrying prey in its bill we used binoculars and cameras to record bird species, number of prey items foraged, prey species, approximate size of prey (estimated relative to bill size), and an observer uncertainty score of prey species identification (out of 100).

### Spatial analysis

**Figure 5:**
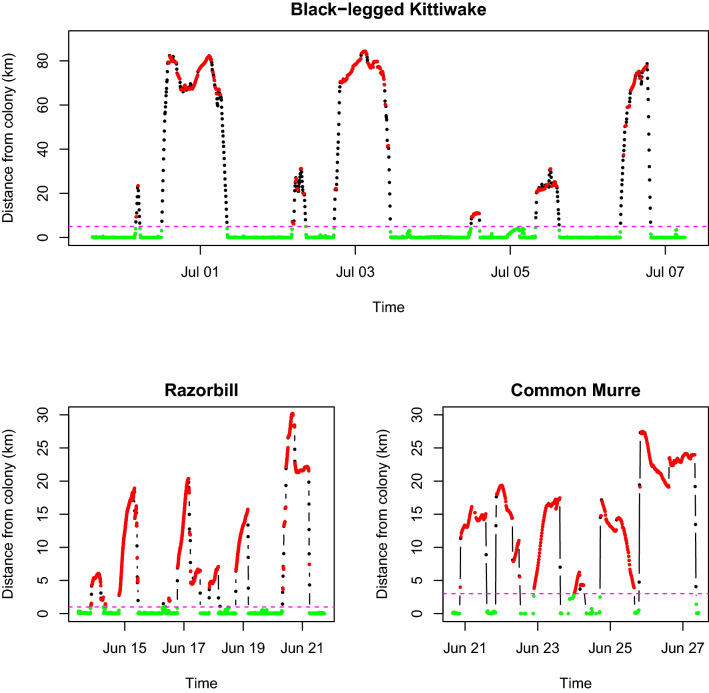
Examples of razorbill, common murre, and black-legged kittiwake GPS activity with all recorded foraging trips. We differentiated between being at the colony (green), flying (black), and foraging (red). The dotted line refers to 1000 m (razorbill), 3000 m (murre), and 5000 m (kittiwake) distance from the colony used to discriminate splashdown from foraging. Note the different scale for kittiwakes.

We excluded both puffins and Île de la Maison from GPS analysis due to insufficient sample sizes and lack of data collected because of GPS malfunction and issues with data recovery. GPS data were analyzed using R software^55^ using the ‘*adehabitatHR*^56^’ and ‘*geosphere*^57^’ packages. The GPS data was cleaned by discarding positions that had fewer than four satellites and/or duplicated positions. To distinguish when foraging behaviour was occurring within each trip and to analyze differences between each species in their trips, we categorized each behaviour as being either at the colony, flying, or foraging. These species splash down in the vicinity of the colony to rest, wash off, or preen in the water^58^. Thus, we assumed that each species was not foraging when they were within:1000 m (razorbills), 3000 m (murres), and 5000 m (kittiwakes) of the colony. This was decided for each species based on the frequency of distances from the colony and how far each species was travelling (Fig. 5). Complete foraging trips were then defined as trips made beyond this splash down with more than four GPS positions.

To estimate a conservative instantaneous speed threshold and discern between flying and foraging behaviour, we used the average flight speed of each tested species: murres, 80 km/h^59,60^, razorbills, 80 km/h^61^, and kittiwakes, 47 km/h^62,63^, and assumed a drift speed of 5 km/h (based on distributions of instantaneous speeds). We calculated a speed threshold for each species:

1 $$Speed\; Threshold = \frac{{2\left( {Drift \;Speed*Average \;Flight \;Speed} \right)}}{Drift \;Speed + Average \;Flight \;Speed}$$

Each species threshold (murres and razorbills: 9.41 km/h, and kittiwakes: 9.04 km/h) was then averaged to a common threshold of 9.29 km/h. We then discriminated that anything equal to or above this speed was considered as flying (Fig. 5).

We computed different foraging trip metrics for each tested species in order to detect interspecific differences of foraging characteristics. This included: maximum distance from the colony (km), trip duration (h), foraging range index (FRI, km), distance at which the first stop was made in a trip (km), the total number of food patches visited within a trip, and the time of day when a bird would depart from the colony to forage. The Foraging Range Index (FRI) of each species provides an estimate of the average distance (km) from the colony that the birds are foraging^64^. A higher FRI indicates that a species travels farther away from the colony to forage than other species. We also calculated a Commuting Index (CI), which reflects degree of commuting within foraging trips:

2 $$CI = 1 - 2*MAD,$$

with MAD being the mean absolute deviance of the relative distance from the colony while birds were considered foraging (below the speed threshold):

3 $$MAD = \frac{1}{n} \mathop \sum \limits_{i = 1}^{n} \left| { X_{i} - \overline{X } } \right| ,$$

where *n* is the total number of GPS positions when foraging in a trip, ${X}_{i}$ a GPS foraging position, and $\stackrel{-}{X}$ the average GPS position while foraging. The relative distance from the colony on a scale between 0 and 1 for each foraging trip; 0 being at the colony and 1 being the furthest point from the colony. This deviation can provide us with an approximation of how much the birds were spending commuting during their trips (CI indexed between 0 and 1; 0 indicating that there was no commuting during the trip, 1 being that all foraging was being done at a specific distance from the colony). FRI, CI, and distances from colony were compared among species using Kruskal–Wallis tests and then subsequently Wilcox-Mann–Whitney tests with Bonferroni Correction^65–67^.

Since we observed multiple successive trips from a single individual, we analyzed all foraging metrics using linear mixed-effect models that were applied using the function ‘*lmer*’ in the ‘*lme4*^68^’ package to overcome issues of pseudo-replication within individuals. Log-transformed foraging parameters were considered as a dependent variable and species were added to the model as fixed factors while bird identity was included as a random factor.

To analyse the intensity of foraging at different locations and the distribution patterns within the foraging range of each bird species we used bivariate normal kernel density analyses (‘*kernelUD*’ function) using the ‘*adehabitatHR 0.4.14*’ package^56^. After transforming data using the kernel function into a raster, we used a smoothing parameter within the same package ‘*h*’ using the ad hoc method ‘*href*’ to obtain utilization distributions (UD) of core range 25, 50, 75, 95% contour polygons within the home range of each tested species^56^. Maps were constructed using ‘ggplot2^69^’, ‘*ggspatial*^70^’, ‘*rnaturalearth*^71^’, ‘*rnaturalearthdata*^72^’ and ‘*sf*^73^’ packages.

We investigated whether there was partitioning within the horizontal foraging dimension by estimating the degree of overlap between each species. We used two different methods within the ‘*kerneloverlaphr*’ function to analyse the degree to which two species share space: (1) the Utilization Distribution Overlap Index (UDOI) and (2) the Bhattacharyya’s Affinity (BA) method^74,75^. The BA (0 signifying no overlap in UDs and 1 identical), and the UDOI (0 signifying no overlap and 1 identical overlap with a uniform distribution) are statistical measures for the degree of similarity amongst UDs and the amount of space-use shared among species, respectively. We used a randomization procedure^75^ to test for statistical differences between the estimated overlap in UD for both measures. This simulation involved pairwise comparisons of the overlap index between species by randomly assigning each species to an individual foraging trip to calculate the random overlap index for both BA and UDOI methods^75^. The simulation then runs 1000 times to obtain a randomized value which can be statistically tested against our observed overlap index using Wilcox-Mann–Whitney tests^66^.

We performed a Principal Components Analysis (PCA) on all seven foraging metrics (departure time of day, duration of foraging trip, number of stops, maximum distance, FRI, distance of first stop, CI) to analyze interspecific discrepancies in foraging space, using ‘*corrplot*’, ‘*FactoMineR*’, and ‘*factoextra*’ packages^76–78^. We analyzed the first two principal components as only those axes had eigenvalues greater than 1. We then conducted a Wilcox-Mann–Whitney test to test the difference between species along the PC1 and PC2 axes^66^.

### Diet analysis

Pictures taken during the feeding observations were analyzed (Supplementary Table S3). Since the colonies are large and the possibility of observing the same bird twice are low, we assumed that there was only one feeding observation per individual. Pictures that were blurry or had a low prey species certainty score (< 50% confidence) were discarded (635 samples kept). We used the following equation to estimate prey size foraged by each seabird species:

4 $$Prey\; length \;\left( {mm} \right) = \frac{{Prey \;length\; \left( {pixels } \right)}}{{Bill \;length\; \left( {pixels} \right)}}*Bill \;length \;\left( {mm} \right)$$

We calculated the average bill length (mm) for each species using our measurements of the culmen (puffin = 49.1 mm $\pm 0.43$; razorbill = 34.3 $\pm$ 0.39 mm; murre = 43.9 mm). We used *ImageJ* to estimate of the prey-to-bill ratio (in pixels)^79^.

We then calculated the frequency of occurrence, numerical abundance and mass energy of prey species for each studied seabird species. Frequency of occurrence refers to the total number of observed individuals bringing back a particular prey species while the numerical abundance is the total number of a prey species items brought back for a given seabird. Further calculations provided us with estimations of the mass of each prey species using equations^40,80–85^. For example, mass of capelin was estimated using the following equation:

5 $$Mass \;of\; capelin = \left( {2.7 \times 10^{ - 5} } \right) \times prey\; length^{2.76} ,$$

where mass is given in g and length in mm^40^^.^ Unknown species were estimated from the average of all species’ mass and larval species mass were assigned a weight of 2 g.

All diet analyses were performed in R 3.6.1 software^55^. To evaluate whether and to what extent the different seabird species were segregated in their diets in terms of loading, we analyzed if they were differing in foraged: (1) prey species, (2) estimated prey size, and/or (3) prey number. The relationship between each bird species (individually and by colony) and prey species were analyzed with ‘*plotly*^86^’ and ‘*dplyr*^87^’ packages. We removed all prey species that had less than five observations to calculate the significance of the relationship between prey and the bird species using χ^2^ squared tests. The relationship between bird species and the number of prey items were analyzed using quasi-Poisson tests^88^. The relationship between the number of prey and prey size for puffins and razorbills was analyzed using a linear regression analysis F-statistic test^89^. The relationship between bird species and prey size was analyzed using Kruskal–Wallis tests and then subsequently Wilcox-Mann–Whitney tests with Bonferroni Correction^65–67^. All figures were then constructed using ‘*ggplot2*^69^’ and ‘*ggsignif*^90^’ packages.

### Ethical statement

All animal experimentation met the Canadian Council for Animal Care wildlife guidelines for ethical treatment of animals (authorization from the Eastern Wildlife Animal Care Committee, Environment and Climate Change Canada; #19RL01). Permits to capture, band, and handle birds were approved by the Bird Banding Office (#10711-D, 10711-G). Access to site were permitted by Parks Canada (#MIN-2019–32098) and Canadian Wildlife Service (access to a Migratory Bird Sanctuary; #RE-70).

## Supplementary Information

Supplementary Information.

## Data Availability

The datasets generated during and analyzed during the current study are available from the corresponding author on request.
