# Supplementary material for: Foraging niche partitioning in sympatric seabird populations
Source: Sci Rep. 2021 Jan 28;11:2493. doi: 10.1038/s41598-021-81583-z (PMC7843985; doi:10.1038/s41598-021-81583-z)
Supplement: Supplementary file 1 — Supplementary Information. [file 41598_2021_81583_MOESM1_ESM.pdf]

FORAGING NICHE PARTITIONING IN SYMPATRIC SEABIRD POPULATIONS

Christina Petalas[1,2, *], Thomas Lazarus[1], Raphael A. Lavoie[3], Kyle H. Elliott[1] and Mélanie F. Guigueno[2]

[1]Department of Natural Resource Sciences, McGill University, Montreal, H3A 0G4, Canada
[2]Department of Biology, McGill University, Montreal, H3A 0G4, Canada
[3]Environment Climate Change Canada, Québec, G1J 0C3, Canada
*christina.petalas@mail.mcgill.ca

Supplementary Information

Supplementary Table S1. Model, weight, number of GPS units installed and recovered, and total number of foraging trips obtained during the 2019 field season. Number of individuals for which data was downloaded from Ecotone devices is shown in parenthesis.

Species	Model of GPS	GPS weight (g)	Installed on BETCH	Installed on IDM	Recovered on BETCH	Recovered on IDM	Number of Trips Recorded (# of individuals)
Razorbill	CatLog2	17	11	2	4	0	43 (4)
Kittiwake	CatLog2	10	23	0	7	0	33 (6)
Murre	Ecotone URIA-SOLAR-300	15.5	16	0	0	0	42 (12)
Puffin	Ecotone URIA-60	6	4	5	1	5	4 (3)

Supplementary Table S2. **(a)** The estimated overlap (utilization distribution overlap index: UDOI) in utilization distributions (UD %s) between three seabird species. (**b)** Estimated overlap (Bhattacharyya's affinity—BA) in utilization distributions (UD %s). *** = p < 0.001. Puffins were excluded from analysis due to small sample size.

A

UD (%)	Black-legged Kittiwake/Razorbill			Black-legged Kittiwake/Common Murre			Razorbill/Common Murre		
	Observed BA	Randomized BA	*p-value*	Observed BA	Randomized BA	*p-value*	Observed BA	Randomized BA	*p-value*
25	0	0.1748	***	0	0.1495	***	0	0.0394	***
50	0	0.3930	***	0.0545	0.3520	***	0.1378	0.3005	***
75	0.0859	0.6278	***	0.3068	0.6183	***	0.2479	0.5572	***
95	0.2576	0.8583	***	0.5291	0.8299	***	0.4036	0.7525	***

B

UD (%)	Black-legged Kittiwake/Razorbill			Black-legged Kittiwake/Common Murre			Razorbill/Common Murre		
	Observed UDOI	Randomized UDOI	*p-value*	Observed UDOI	Randomized UDOI	*p - value*	Observed UDOI	Randomized UDOI	*p-value*
25	0	0.0317	***	0	0.0268	***	0	0.0082	***
50	0	0.1705	***	0.003	0.1338	***	0.019	0.0999	***
75	0.008	0.5253	***	0.106	0.4437	***	0.062	0.3920	***
95	0.088	1.3893	***	0.386	1.0593	***	0.173	1.2083	***

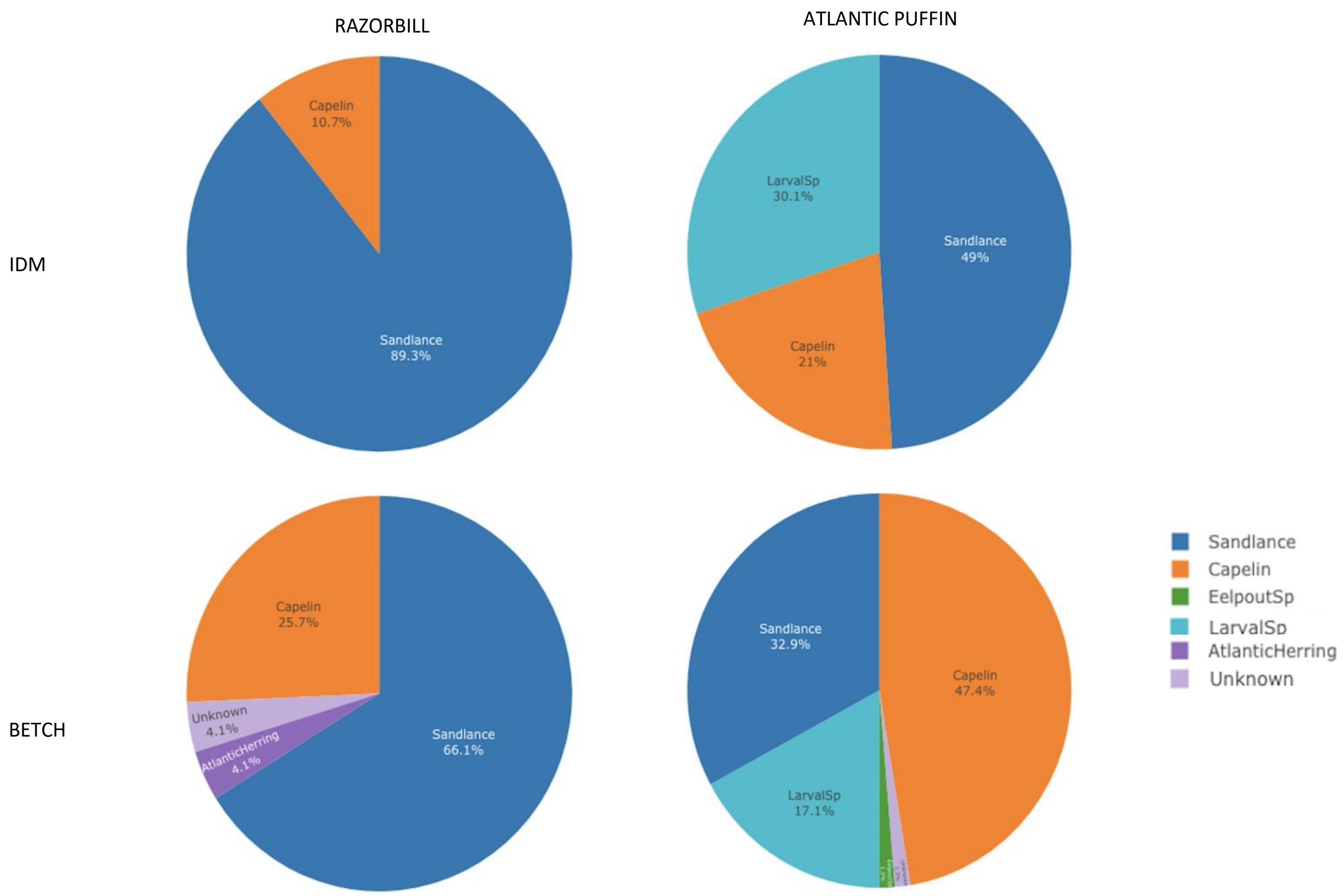

Supplementary Figure 1. Frequency of occurrence of prey species foraged on each island. Top row corresponds to loaded prey species on Île de la Maison (IDM) for Razorbills (left) and Atlantic Puffins (right). Bottom row corresponds to the prey species foraged on Betchouanes (BETCH).

Supplementary Table 3. All feeding watches conducted on both islands. Île de la Maison (IDM) and Betchouanes (BETCH). During the feeding watches either data was collected by observation, photograph, or a combination of both. Data included in this table were after discarding all unknown and/or unidentifiable pictures.

	**Feeding Watch**							
	**01-Jul**	**04-Jul**	**05-Jul**	**06-Jul**	**09-Jul**	**10-Jul**	**11-Jul**	**12-Jul**
**Island**	IDM	IDM	BETCH	IDM	BETCH	BETCH	BETCH	IDM
Observation only	1	6	7	25	2	10	33	7
Photograph only	0	2	0	0	65	143	195	43
Observation and photograph	0	16	35	44	0	0	0	0
**Total**	**1**	**24**	**42**	**69**	**67**	**153**	**228**	**50**
